# Supplementary material for: Parasite DNA and Markers of Decreased Immune Activation Associate Prospectively with Cardiac Functional Decline over 10 Years among Trypanosoma cruzi Seropositive Individuals in Brazil
Source: Int J Mol Sci. 2023 Dec 19;25(1):44. doi: 10.3390/ijms25010044 (PMC10779141; doi:10.3390/ijms25010044)
Supplement: Supplementary file 1 [file ijms-25-00044-s001.zip › ijms-2627141-supplementary.pdf]

Supplementary Figure S1: CONSORT diagram

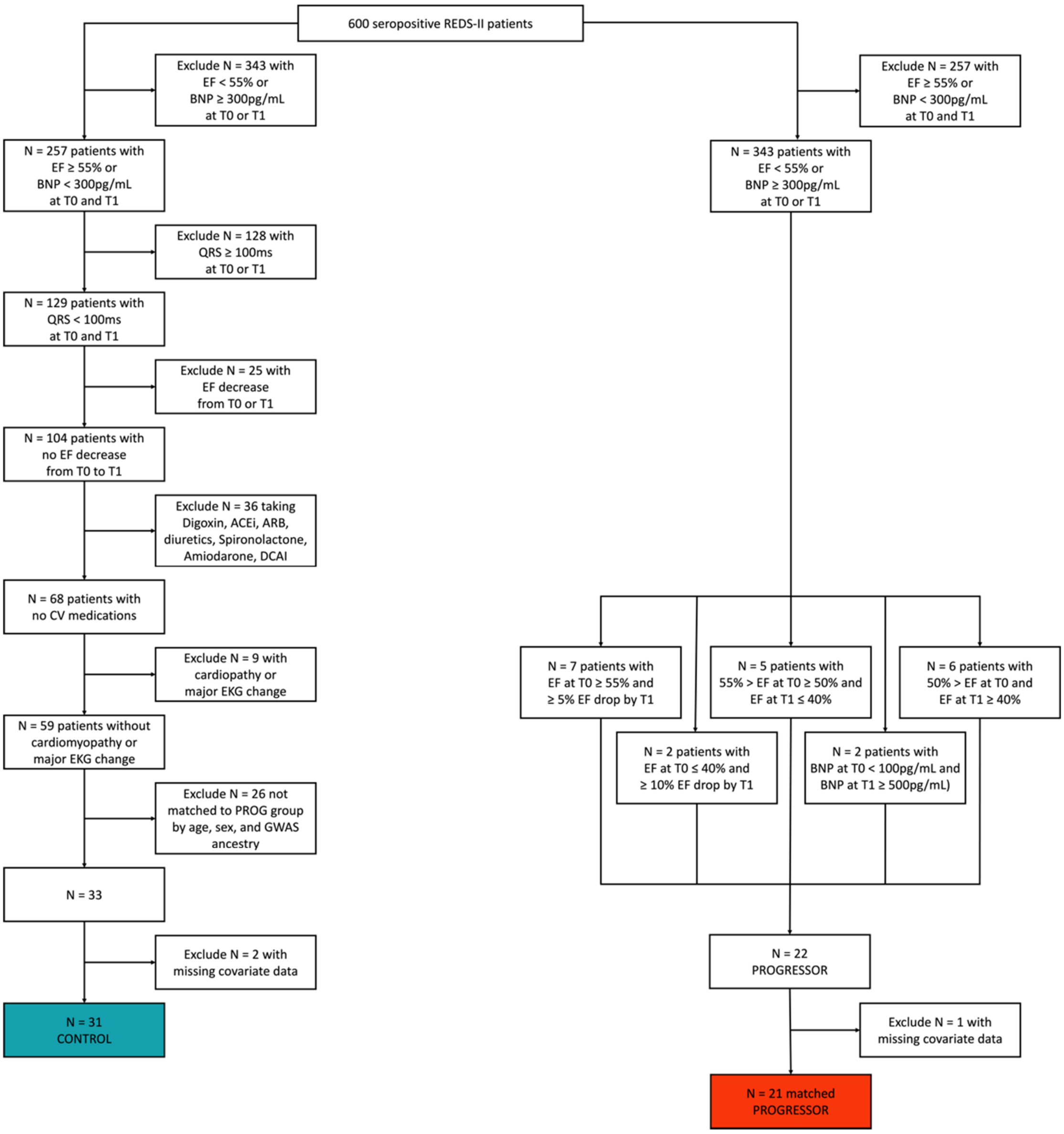

Supplementary Table S1: Full list of normalized protein expression levels

| **Protein** | **CONTROL N = 31** | **PROGRESSORS N = 21** | **P value** |
|---|---|---|---|
| IL12RB1 | 0.42 (0.47) | 0.27 (0.60) | 0.322 |
| IL1RL2 | 0.47 (0.64) | 0.18 (0.35) | 0.065 |
| IL1A | 0.14 (0.99) | 0.38 (1.13) | 0.424 |
| GBP2 | 1.79 (1.91) | 0.82 (1.93) | 0.078 |
| FGF5 | -0.52 (0.92) | -1.56 (1.26) | 0.001 |
| IFNG | 0.64 (1.17) | 0.54 (0.89) | 0.733 |
| SLAMF1 | 0.78 (0.75) | 0.35 (0.85) | 0.061 |
| ENAH | -0.87 (1.55) | -1.56 (1.29) | 0.101 |
| IL15RA | 0.32 (0.58) | 0.26 (0.76) | 0.757 |
| MVK | -0.53 (1.24) | -1.07 (1.50) | 0.165 |
| WNT9A | -1.73 (0.39) | -1.93 (0.36) | 0.068 |
| CSF3 | 0.98 (0.66) | 0.85 (0.85) | 0.531 |
| AMN | -0.87 (0.47) | -1.29 (0.66) | 0.010 |
| ITGB6 | -0.38 (0.75) | -0.39 (0.45) | 0.963 |
| JCHAIN | 0.92 (0.58) | 1.55 (0.70) | 0.001 |
| IRAK1 | 1.23 (1.47) | 0.83 (1.86) | 0.393 |
| IL2 | 0.25 (0.41) | 0.20 (0.48) | 0.693 |
| IL10RA | 0.41 (1.45) | 0.54 (1.42) | 0.749 |
| LRRN1 | 1.72 (0.68) | 2.10 (0.96) | 0.100 |
| PREB | 0.83 (1.07) | 1.00 (2.05) | 0.690 |
| IL24 | 0.16 (0.58) | 0.13 (0.56) | 0.850 |
| CEP164 | 1.19 (1.27) | 1.18 (1.48) | 0.976 |
| NBN | 0.25 (2.10) | -0.74 (1.77) | 0.082 |
| FCRL3 | 0.21 (0.58) | 0.21 (0.80) | 0.985 |
| PRKAB1 | -0.42 (0.93) | -0.53 (1.14) | 0.683 |
| PNPT1 | 1.24 (2.26) | 0.25 (2.21) | 0.125 |
| ARTN | -0.39 (0.52) | -0.13 (1.99) | 0.478 |
| RABGAP1L | 1.24 (0.98) | 0.95 (1.38) | 0.367 |
| NCLN | 0.17 (0.47) | 0.43 (0.68) | 0.111 |
| IL22RA1 | 0.45 (0.59) | 0.53 (0.66) | 0.650 |
| IL2RB | 0.07 (0.38) | 0.06 (0.46) | 0.967 |
| MYO9B | 0.63 (1.39) | -0.04 (1.62) | 0.115 |
| AOC1 | 0.16 (0.84) | -0.21 (0.76) | 0.114 |
| IL20 | -1.18 (0.57) | -1.17 (0.53) | 0.948 |
| RGS8 | 0.64 (0.63) | 1.05 (1.23) | 0.118 |
| IL11 | -0.23 (0.57) | -0.10 (0.57) | 0.409 |
| IL17F | -0.15 (0.95) | -0.32 (0.60) | 0.474 |
| SCGN | 0.13 (0.49) | 0.40 (0.68) | 0.094 |
| CXCL14 | -0.87 (0.74) | -1.21 (0.62) | 0.095 |
| IL3RA | 0.46 (0.45) | 0.29 (0.55) | 0.214 |
| SIT1 | 0.58 (1.15) | 0.07 (1.00) | 0.105 |
| FXYD5 | 0.35 (0.80) | 0.02 (0.68) | 0.127 |
| CXCL12 | 1.50 (1.32) | 0.04 (1.28) | <0.001 |
| NRTN | 0.25 (0.60) | 0.21 (0.53) | 0.790 |
| DGKZ | 0.24 (0.50) | 0.25 (0.74) | 0.992 |
| PADI2 | 1.28 (1.20) | 2.32 (2.23) | 0.034 |
| PRKCQ | 0.17 (0.78) | 0.16 (1.04) | 0.987 |
| IL17A | -0.11 (0.72) | -0.18 (0.32) | 0.676 |
| NFATC3 | -1.64 (0.44) | -1.38 (0.57) | 0.072 |
| GALNT3 | 0.81 (0.85) | 0.44 (0.66) | 0.106 |
| IL5 | -5.47 (1.84) | -5.40 (2.38) | 0.901 |
| TNF | -0.53 (0.81) | -1.12 (0.68) | 0.008 |
| TANK | 1.00 (0.99) | 0.55 (0.72) | 0.078 |
| SPRY2 | 2.02 (2.23) | 0.83 (1.64) | 0.041 |
| TPT1 | 1.99 (1.18) | 1.53 (1.12) | 0.169 |
| IL17C | -1.14 (1.04) | -1.78 (0.61) | 0.014 |
| YTHDF3 | 1.84 (2.23) | 1.24 (1.70) | 0.302 |
| WAS | 3.14 (2.42) | 1.11 (1.83) | 0.002 |
| TNFRSF13C | 0.18 (0.65) | -0.05 (0.53) | 0.190 |
| IL17D | -0.24 (0.68) | -0.78 (0.78) | 0.010 |
| MILR1 | -1.46 (0.64) | -1.92 (0.46) | 0.007 |

| | | | |
|---|---|---|---|
| PRDX3 | 2.47 (2.20) | 1.31 (2.26) | 0.070 |
| ICA1 | 1.73 (1.37) | 1.11 (1.77) | 0.162 |
| PAPPA | 0.71 (0.80) | 0.26 (0.78) | 0.049 |
| IL20RA | -0.10 (0.57) | -0.07 (0.88) | 0.860 |
| EIF5A | 0.53 (0.58) | 1.28 (0.72) | <0.001 |
| JUN | -0.24 (0.68) | -0.30 (0.67) | 0.746 |
| RAB37 | 1.96 (2.00) | 1.74 (2.50) | 0.716 |
| IL4 | 0.65 (0.93) | 0.55 (1.17) | 0.746 |
| IL1B | 1.39 (1.06) | 1.41 (1.35) | 0.949 |
| IL33 | 0.93 (0.67) | 0.83 (0.53) | 0.552 |
| LILRB4 | -0.82 (0.63) | -1.04 (0.50) | 0.184 |
| IL13 | 0.26 (0.47) | 0.35 (0.49) | 0.497 |
| IL10 | -6.43 (0.82) | -6.55 (0.99) | 0.627 |
| ARNT | -1.96 (0.45) | -2.15 (0.51) | 0.150 |
| TNFAIP8 | 1.49 (1.55) | 0.85 (0.92) | 0.093 |
| LTO1 | 0.42 (0.40) | 0.63 (0.48) | 0.105 |
| ACTN4 | 2.67 (1.79) | 2.13 (2.17) | 0.334 |
| LAP3 | 3.33 (1.84) | 2.09 (1.86) | 0.022 |
| AMBN | 0.05 (0.27) | 0.11 (0.28) | 0.424 |
| SPINK4 | 1.09 (0.93) | 1.16 (0.85) | 0.786 |
| NUB1 | 2.01 (1.97) | 1.65 (2.30) | 0.547 |
| PSPN | -0.92 (1.73) | -1.33 (1.94) | 0.429 |
| ALDH3A1 | -0.01 (1.03) | -0.62 (0.89) | 0.033 |
| BCL2L11 | -1.99 (0.57) | -2.33 (0.63) | 0.051 |
| SH2D1A | -0.09 (1.31) | -0.71 (1.31) | 0.099 |
| FOXO1 | 1.40 (1.47) | 0.53 (1.27) | 0.031 |
| VASH1 | 0.15 (0.64) | -0.07 (0.27) | 0.148 |
| BID | 4.28 (1.63) | 4.17 (1.61) | 0.824 |
| METAP1D | 2.72 (2.60) | 1.55 (2.64) | 0.122 |
| HLA.DRA | 3.14 (1.47) | 2.11 (1.32) | 0.013 |
| TBC1D5 | 2.31 (1.80) | 1.87 (2.00) | 0.408 |
| EPO | -0.25 (0.88) | 0.05 (1.79) | 0.427 |
| CCL7 | 0.85 (0.84) | 0.70 (0.52) | 0.484 |
| DAPP1 | 3.13 (2.96) | 1.35 (2.72) | 0.033 |
| EDAR | 1.00 (1.18) | 0.78 (1.13) | 0.505 |
| TRIM5 | 2.02 (1.57) | 2.08 (2.09) | 0.905 |
| DPP10 | 0.64 (0.47) | 0.60 (0.52) | 0.797 |
| ITGA6 | 4.32 (1.59) | 2.90 (1.71) | 0.004 |
| FCRL6 | 0.86 (1.06) | 0.57 (0.65) | 0.263 |
| LSP1 | -0.01 (1.36) | -0.36 (1.28) | 0.357 |
| STX8 | 0.39 (1.80) | -0.12 (1.93) | 0.337 |
| HLA.E | -0.31 (0.60) | -0.57 (1.01) | 0.260 |
| TRIM21 | 2.94 (1.49) | 2.25 (2.11) | 0.170 |
| PSIP1 | 0.41 (2.08) | -0.85 (2.31) | 0.046 |
| IL7 | 2.03 (0.90) | 1.78 (1.11) | 0.376 |
| SAMD9L | 3.14 (1.91) | 2.33 (2.18) | 0.162 |
| SIGLEC10 | 0.29 (0.51) | 0.35 (0.66) | 0.711 |
| ISM1 | -2.34 (0.89) | -3.38 (0.80) | <0.001 |
| SLC39A5 | -0.48 (0.63) | -0.86 (0.72) | 0.049 |
| BTN3A2 | 0.97 (0.49) | 0.65 (0.82) | 0.090 |
| MLN | -1.62 (0.97) | -1.77 (0.74) | 0.556 |
| SCRN1 | 4.66 (1.84) | 3.23 (1.80) | 0.008 |
| PROK1 | 0.47 (0.95) | 0.10 (0.87) | 0.157 |
| IKBKG | 2.03 (1.91) | 1.37 (2.38) | 0.275 |
| NFATC1 | 1.34 (1.50) | 1.12 (2.09) | 0.659 |
| CCL26 | 2.64 (1.64) | 1.58 (1.42) | 0.019 |
| CLEC4C | 0.34 (0.76) | 0.27 (0.65) | 0.724 |
| CEACAM21 | -0.04 (1.07) | -0.10 (1.08) | 0.859 |
| PSMG3 | 1.41 (1.85) | 0.07 (1.67) | 0.010 |
| NCF2 | 1.41 (2.79) | 0.16 (2.48) | 0.106 |
| NPPC | -4.15 (1.56) | -5.51 (2.21) | 0.012 |
| CCL28 | 0.85 (1.00) | 0.15 (0.83) | 0.011 |
| PTX3 | 1.17 (0.74) | 0.93 (0.73) | 0.246 |
| PTH1R | -2.48 (0.76) | -2.52 (0.85) | 0.850 |
| EGLN1 | -0.60 (1.88) | -1.59 (1.48) | 0.047 |
| CLEC4A | 0.47 (0.57) | 0.78 (0.63) | 0.068 |
| OSM | 1.67 (1.11) | 1.57 (1.43) | 0.787 |

| | | | |
|---|---|---|---|
| HSD11B1 | 1.03 (0.96) | 0.28 (1.07) | 0.011 |
| IL4R | 0.49 (0.37) | 0.73 (0.42) | 0.037 |
| IRAK4 | 3.83 (2.30) | 2.84 (2.58) | 0.155 |
| ICAM4 | 0.51 (0.73) | 0.30 (0.48) | 0.254 |
| DECR1 | 2.17 (2.20) | 1.54 (3.12) | 0.394 |
| LY75 | 0.45 (0.48) | 0.33 (0.47) | 0.366 |
| ITGA11 | 1.12 (0.50) | 1.25 (0.39) | 0.311 |
| AXIN1 | 1.80 (2.45) | 0.51 (1.98) | 0.050 |
| ANXA11 | 2.35 (1.94) | 1.40 (1.87) | 0.087 |
| CD4 | -0.45 (0.79) | -1.21 (0.79) | 0.001 |
| IL17RB | -0.24 (0.77) | -0.05 (0.67) | 0.349 |
| LTA | 0.87 (0.49) | 0.32 (0.59) | 0.001 |
| PIK3AP1 | 3.57 (1.71) | 2.43 (1.61) | 0.019 |
| MGMT | 3.65 (2.50) | 2.00 (2.49) | 0.024 |
| HEXIM1 | 0.93 (2.04) | -0.64 (1.48) | 0.004 |
| CD200 | 0.54 (0.51) | 0.50 (0.39) | 0.766 |
| GZMB | 0.44 (2.27) | -0.40 (2.83) | 0.244 |
| IL32 | 0.11 (0.64) | 0.09 (0.51) | 0.932 |
| LIFR | 0.18 (0.47) | -0.22 (0.36) | 0.002 |
| CD84 | -0.53 (0.74) | -1.15 (0.76) | 0.005 |
| RAB6A | 5.31 (2.18) | 3.32 (2.85) | 0.006 |
| CLEC4D | 0.21 (1.03) | -0.16 (1.42) | 0.286 |
| CCL3 | 0.68 (0.68) | 0.82 (1.80) | 0.677 |
| PARP1 | 2.71 (2.27) | 1.88 (2.30) | 0.207 |
| FABP9 | 0.74 (0.95) | 0.43 (0.68) | 0.212 |
| CLEC4G | 0.01 (0.50) | -0.12 (0.46) | 0.345 |
| NTF3 | -0.90 (1.04) | -1.08 (0.59) | 0.458 |
| TNFSF11 | 0.30 (1.02) | 0.19 (0.77) | 0.670 |
| MICB | -0.52 (1.74) | -0.16 (1.51) | 0.444 |
| BANK1 | 2.42 (2.59) | 1.27 (2.51) | 0.118 |
| CD200R1 | 0.54 (0.51) | 0.34 (0.46) | 0.154 |
| KRT19 | -0.23 (0.72) | -0.18 (0.80) | 0.825 |
| PLXNA4 | 1.52 (1.54) | 1.34 (1.72) | 0.707 |
| PTPRM | 0.42 (0.45) | 0.21 (0.36) | 0.081 |
| CD70 | 1.21 (0.73) | 1.02 (0.64) | 0.327 |
| TGFA | 0.68 (0.98) | 0.45 (0.52) | 0.337 |
| IL5RA | 0.40 (0.78) | 0.10 (0.82) | 0.178 |
| SLAMF7 | 1.45 (1.01) | 1.26 (0.56) | 0.430 |
| GOPC | 2.18 (1.98) | 0.66 (1.65) | 0.006 |
| MAP2K6 | 1.33 (2.55) | 0.31 (2.21) | 0.142 |
| NT5C3A | 3.53 (2.51) | 1.94 (2.61) | 0.032 |
| ARHGEF12 | 1.01 (1.65) | 0.54 (2.02) | 0.369 |
| CASP2 | 1.28 (1.60) | 0.99 (2.00) | 0.570 |
| SRPK2 | 1.28 (1.45) | 0.80 (1.69) | 0.278 |
| FGF2 | 0.87 (1.15) | 0.47 (0.86) | 0.186 |
| ITM2A | -0.32 (0.47) | -0.41 (0.33) | 0.411 |
| BACH1 | 1.34 (1.40) | 0.77 (1.78) | 0.196 |
| IFNLR1 | 0.08 (0.49) | 0.14 (0.35) | 0.594 |
| TRAF2 | 0.90 (1.24) | 0.57 (1.57) | 0.401 |
| SULT2A1 | 3.04 (1.37) | 2.24 (1.41) | 0.048 |
| COL9A1 | -0.02 (0.65) | -0.18 (0.48) | 0.336 |
| CNTNAP2 | 0.71 (0.62) | 0.54 (0.51) | 0.296 |
| MAPK9 | 3.70 (1.19) | 3.29 (2.27) | 0.396 |
| BCR | 1.37 (2.00) | -0.12 (1.70) | 0.007 |
| CLIP2 | 4.27 (2.76) | 3.32 (2.84) | 0.230 |
| SELPLG | 0.03 (0.45) | -0.01 (0.48) | 0.740 |
| CXADR | 0.97 (0.64) | 1.04 (0.65) | 0.708 |
| IL15 | 0.30 (0.45) | 0.30 (0.44) | 0.949 |
| IL6 | 0.83 (1.16) | 0.82 (0.76) | 0.985 |
| FCAR | 0.91 (0.74) | 1.06 (1.00) | 0.542 |
| CD83 | 0.91 (0.52) | 0.86 (0.41) | 0.750 |
| NCR1 | 0.33 (0.51) | 0.05 (0.52) | 0.058 |
| TNFSF10 | -0.61 (0.61) | -0.64 (0.45) | 0.837 |
| TLR3 | 0.59 (0.76) | 0.56 (0.52) | 0.836 |
| CXCL6 | 2.64 (1.08) | 1.65 (1.98) | 0.025 |
| PCDH1 | -0.70 (0.34) | -0.90 (0.24) | 0.028 |
| HPCAL1 | 0.17 (2.19) | -1.78 (1.81) | 0.001 |

| | | | |
|---|---|---|---|
| CD40LG | 1.15 (1.71) | 0.74 (1.49) | 0.373 |
| CKAP4 | 0.06 (1.38) | -1.03 (2.39) | 0.042 |
| FKBP1B | 3.09 (3.02) | 0.86 (2.39) | 0.007 |
| IDS | 2.65 (0.74) | 3.29 (0.61) | 0.002 |
| DFFA | 1.21 (1.89) | -0.41 (2.20) | 0.007 |
| TGFB1 | 0.80 (0.90) | 0.70 (1.13) | 0.724 |
| CXCL17 | -2.01 (1.48) | -3.40 (1.50) | 0.002 |
| NUDC | 1.88 (1.42) | 0.52 (2.30) | 0.011 |
| TNFSF12 | -0.47 (1.39) | -1.72 (0.94) | 0.001 |
| EIF4G1 | 4.33 (2.28) | 3.22 (2.15) | 0.086 |
| BSG | 1.37 (0.47) | 1.11 (0.39) | 0.041 |
| DNAJA2 | 3.74 (1.94) | 3.06 (2.07) | 0.233 |
| CD244 | 0.98 (0.56) | 0.88 (0.62) | 0.561 |
| KLRB1 | 0.61 (0.62) | 0.46 (0.48) | 0.357 |
| SERPINB8 | 0.04 (1.49) | -0.16 (1.51) | 0.647 |
| CXCL8 | 1.42 (1.46) | 1.08 (1.43) | 0.400 |
| KLRD1 | 0.47 (0.69) | 0.15 (0.48) | 0.069 |
| IL16 | 1.05 (1.22) | 0.00 (1.85) | 0.017 |
| NFASC | 0.62 (0.49) | 0.68 (0.40) | 0.612 |
| CD79B | -1.17 (0.80) | -1.72 (0.62) | 0.011 |
| CLEC7A | 0.26 (0.60) | 0.20 (0.71) | 0.760 |
| CD22 | 0.65 (0.58) | 0.75 (0.53) | 0.518 |
| LAMP3 | -0.38 (0.58) | -0.43 (0.85) | 0.828 |
| FCRL2 | 0.40 (0.83) | -0.02 (0.53) | 0.049 |
| LAT | 2.75 (2.46) | 1.25 (2.34) | 0.033 |
| GMPR | 2.04 (1.84) | 0.09 (1.74) | <0.001 |
| TPSAB1 | -0.83 (0.49) | -0.53 (0.60) | 0.054 |
| PPP1R9B | 2.88 (2.37) | 1.02 (2.06) | 0.005 |
| PRDX5 | 1.79 (2.11) | -0.79 (1.84) | <0.001 |
| ADA | -2.74 (1.29) | -3.13 (1.08) | 0.257 |
| TNFRSF11A | 0.49 (0.56) | 0.38 (0.51) | 0.457 |
| CD160 | 0.02 (0.87) | -0.56 (0.67) | 0.013 |
| HCLS1 | 0.81 (2.56) | -1.69 (1.71) | <0.001 |
| CD6 | 0.69 (0.91) | 0.14 (0.61) | 0.019 |
| VEGFA | 0.87 (1.17) | 0.67 (0.97) | 0.519 |
| ADAM23 | -0.63 (1.11) | -1.84 (1.58) | 0.002 |
| IL18R1 | 0.44 (0.51) | 0.50 (0.41) | 0.676 |
| TNFRSF4 | 0.29 (0.48) | 0.13 (0.42) | 0.221 |
| C1QA | -0.11 (0.39) | -0.56 (0.87) | 0.013 |
| CCL13 | 1.24 (1.18) | 0.46 (1.35) | 0.033 |
| HGF | 0.57 (0.74) | 0.52 (0.54) | 0.783 |
| MERTK | -0.12 (0.51) | -0.24 (0.27) | 0.334 |
| AGRP | 0.02 (1.40) | -1.75 (1.17) | <0.001 |
| CTSO | -0.39 (0.61) | -0.56 (0.46) | 0.270 |
| FLT3LG | 0.24 (0.50) | 0.20 (0.46) | 0.769 |
| VEGFD | 1.39 (0.64) | 1.44 (0.48) | 0.748 |
| GZMA | 0.34 (0.78) | 0.39 (1.03) | 0.840 |
| CLSTN2 | -0.58 (0.66) | -1.14 (0.81) | 0.008 |
| FASLG | 0.45 (0.63) | 0.20 (0.50) | 0.125 |
| IL12B | 0.05 (0.76) | -0.25 (0.54) | 0.131 |
| CDSN | -0.08 (0.35) | -0.33 (0.27) | 0.007 |
| CCL11 | -0.52 (1.29) | -2.11 (2.37) | 0.003 |
| KYNU | -2.14 (1.08) | -3.28 (1.07) | <0.001 |
| LY9 | 0.57 (0.55) | 0.25 (0.43) | 0.028 |
| CCL20 | 0.45 (0.98) | -0.02 (0.70) | 0.066 |
| MMP1 | 0.09 (1.98) | -0.46 (1.87) | 0.320 |
| PGF | -0.12 (0.40) | -0.26 (0.40) | 0.239 |
| CCL25 | -0.12 (1.04) | -1.65 (1.98) | 0.001 |
| PKLR | 3.23 (1.16) | 2.60 (1.32) | 0.078 |
| NCK2 | 1.58 (1.69) | 0.39 (0.93) | 0.005 |
| TIMP3 | 2.82 (2.05) | 1.64 (1.81) | 0.039 |
| PLA2G4A | 1.87 (1.91) | 0.96 (1.84) | 0.093 |
| CCL21 | -0.62 (0.54) | -1.08 (0.55) | 0.004 |
| MMP10 | 0.07 (0.64) | 0.15 (0.70) | 0.666 |
| SCGB1A1 | 0.57 (0.74) | 0.39 (0.71) | 0.387 |
| ENPP7 | -0.76 (1.55) | -1.58 (1.69) | 0.079 |
| SIGLEC1 | 0.61 (0.60) | 0.50 (0.46) | 0.507 |

| | | | |
|---|---|---|---|
| F2R | -1.27 (1.52) | -2.71 (1.42) | 0.001 |
| CD48 | 0.43 (0.47) | 0.30 (0.40) | 0.309 |
| CCL23 | 0.88 (0.74) | 0.68 (0.55) | 0.296 |
| CRLF1 | -0.71 (0.43) | -0.66 (0.33) | 0.619 |
| IL1RN | 0.52 (1.19) | 0.26 (1.07) | 0.415 |
| CRIM1 | 0.28 (0.42) | 0.16 (0.37) | 0.266 |
| TNFRSF13B | 1.10 (0.64) | 0.84 (0.43) | 0.114 |
| ANGPTL4 | -1.48 (1.30) | -1.97 (1.39) | 0.199 |
| CST7 | 1.28 (1.60) | 1.59 (1.34) | 0.471 |
| ERBB3 | 0.67 (0.42) | 0.53 (0.24) | 0.162 |
| NELL2 | 0.51 (0.39) | 0.54 (0.42) | 0.778 |
| MANF | 4.73 (3.17) | 3.00 (2.82) | 0.050 |
| LY6D | 0.61 (0.60) | 0.70 (0.57) | 0.597 |
| CCN2 | 1.44 (0.98) | 0.89 (0.76) | 0.036 |
| GLOD4 | 2.03 (0.93) | 1.62 (0.94) | 0.133 |
| MGLL | 2.54 (2.37) | 1.29 (2.23) | 0.062 |
| DNER | 0.65 (0.44) | 0.48 (0.33) | 0.148 |
| BTN2A1 | 0.44 (0.36) | 0.38 (0.45) | 0.598 |
| SHMT1 | 1.04 (1.64) | -0.04 (1.94) | 0.036 |
| FGF19 | -0.25 (0.87) | -0.87 (1.47) | 0.065 |
| CD58 | 0.42 (0.30) | 0.22 (0.33) | 0.026 |
| PTPN6 | 1.82 (2.33) | -0.05 (1.72) | 0.003 |
| HSPA1A | 2.65 (1.96) | 1.37 (2.15) | 0.032 |
| CSF1 | 0.21 (0.39) | 0.25 (0.44) | 0.727 |
| SPINT2 | -2.10 (0.81) | -2.80 (0.73) | 0.002 |
| GAL | -0.09 (1.54) | -1.75 (0.63) | <0.001 |
| SCG3 | -0.79 (0.90) | -1.74 (0.92) | 0.001 |
| PDLIM7 | 4.12 (3.36) | 2.25 (3.26) | 0.052 |
| SMOC2 | 0.23 (0.67) | -0.03 (0.53) | 0.148 |
| TREM2 | 1.24 (0.87) | 1.26 (0.61) | 0.930 |
| MZB1 | 1.27 (0.71) | 1.31 (0.46) | 0.822 |
| TNFSF13 | 0.02 (0.83) | -0.98 (1.22) | 0.001 |
| ROBO1 | 0.47 (0.46) | 0.25 (0.29) | 0.056 |
| TNFRSF11B | 0.32 (0.49) | 0.31 (0.47) | 0.938 |
| ENPP5 | -0.74 (0.56) | -1.38 (0.86) | 0.002 |
| LAIR1 | 0.53 (0.81) | 0.51 (0.53) | 0.936 |
| COLEC12 | -0.74 (1.47) | -2.13 (1.41) | 0.001 |
| SIRPB1 | 0.61 (0.58) | 0.39 (0.73) | 0.249 |
| ANGPT1 | 2.67 (1.48) | 1.98 (1.28) | 0.089 |
| PDGFB | 3.77 (1.72) | 3.49 (1.53) | 0.557 |
| CRKL | 3.80 (2.64) | 2.41 (2.64) | 0.069 |
| EPCAM | -0.10 (0.83) | 0.37 (1.06) | 0.080 |
| DNPH1 | 3.27 (1.29) | 2.44 (1.50) | 0.038 |
| CCL17 | 2.62 (1.29) | 1.96 (1.36) | 0.080 |
| MEGF10 | 0.66 (0.54) | 0.43 (0.38) | 0.097 |
| CRHBP | 0.68 (0.54) | 0.46 (0.88) | 0.269 |
| LGALS4 | -0.27 (0.62) | -0.25 (0.64) | 0.906 |
| LHPP | 2.19 (1.20) | 1.93 (1.19) | 0.455 |
| TPP1 | 0.03 (1.27) | -0.95 (1.54) | 0.015 |
| CRELD2 | 1.47 (1.06) | 1.06 (0.92) | 0.155 |
| CTRC | 0.94 (0.92) | 0.49 (0.60) | 0.060 |
| MEPE | -3.78 (1.07) | -4.82 (0.79) | <0.001 |
| CDON | -0.50 (0.42) | -0.91 (0.50) | 0.002 |
| ADGRE2 | 0.72 (0.38) | 0.85 (0.42) | 0.231 |
| AGER | 0.20 (0.62) | -0.17 (0.69) | 0.045 |
| IL1R2 | 0.39 (0.33) | 0.36 (0.24) | 0.741 |
| ESM1 | 1.93 (0.49) | 1.92 (0.40) | 0.949 |
| SPON1 | -0.05 (0.61) | -0.19 (0.47) | 0.376 |
| ATP5IF1 | 3.06 (3.38) | -0.05 (2.77) | 0.001 |
| SKAP2 | 4.16 (3.00) | 2.55 (3.12) | 0.067 |
| CXCL1 | 2.97 (1.48) | 2.47 (2.05) | 0.314 |
| PRSS8 | 0.20 (0.63) | 0.19 (0.75) | 0.933 |
| PLAUR | -0.42 (0.80) | -0.73 (0.70) | 0.158 |
| CCL22 | 2.13 (0.95) | 1.74 (0.61) | 0.103 |
| PRELP | -1.69 (1.19) | -3.05 (1.78) | 0.002 |
| MATN2 | -0.28 (0.59) | -0.62 (0.46) | 0.031 |
| LTBR | 0.43 (0.42) | 0.36 (0.42) | 0.557 |

| | | | |
|---|---|---|---|
| LAMA4 | -0.01 (0.93) | -0.44 (0.67) | 0.078 |
| FST | 0.32 (0.84) | 0.26 (0.92) | 0.831 |
| CHRDL1 | -0.41 (0.75) | -0.54 (0.61) | 0.517 |
| CCL24 | 0.95 (0.97) | 0.86 (1.07) | 0.758 |
| LGMN | -0.20 (0.80) | -1.00 (0.66) | <0.001 |
| DAG1 | 1.10 (1.12) | 0.52 (1.09) | 0.070 |
| PNLIPRP2 | -0.50 (2.74) | -2.49 (4.16) | 0.042 |
| OSCAR | 0.58 (0.55) | 0.30 (0.58) | 0.087 |
| PON3 | 0.08 (0.29) | 0.11 (0.37) | 0.790 |
| FABP1 | -0.37 (1.58) | -1.90 (2.32) | 0.007 |
| MPIG6B | 1.59 (2.10) | 0.95 (2.08) | 0.286 |
| B4GALT1 | -1.54 (0.77) | -2.11 (0.56) | 0.005 |
| LGALS9 | 0.55 (0.56) | 0.56 (0.54) | 0.920 |
| FSTL3 | 0.36 (0.57) | 0.31 (0.53) | 0.766 |
| TNFRSF14 | 0.53 (0.54) | 0.48 (0.58) | 0.720 |
| REG4 | 0.19 (0.68) | 0.39 (0.78) | 0.313 |
| WFIKKN2 | 0.23 (0.96) | -0.50 (0.86) | 0.007 |
| AGRN | 0.96 (0.59) | 0.86 (0.48) | 0.537 |
| TFF2 | 1.37 (0.61) | 1.25 (0.75) | 0.517 |
| CXCL3 | 4.15 (1.60) | 3.53 (1.83) | 0.202 |
| NME3 | -0.16 (0.43) | -0.42 (0.70) | 0.099 |
| CKMT1A | -0.64 (1.40) | -1.43 (1.43) | 0.055 |
| FIS1 | 1.96 (1.81) | 1.31 (1.69) | 0.201 |
| SCGB3A2 | 1.42 (1.06) | 1.33 (0.98) | 0.746 |
| CD40 | 0.84 (1.07) | 1.12 (1.45) | 0.433 |
| OMD | -1.72 (0.40) | -1.99 (0.51) | 0.040 |
| ANGPTL2 | 1.00 (0.55) | 0.68 (0.67) | 0.069 |
| IL18 | 0.09 (0.76) | -0.16 (0.84) | 0.262 |
| CCL4 | 1.01 (0.80) | 0.61 (0.82) | 0.090 |
| IL10RB | 0.48 (0.44) | 0.14 (0.53) | 0.016 |
| CXCL10 | 1.70 (1.19) | 0.63 (1.90) | 0.016 |
| EGF | 2.74 (2.38) | 2.02 (2.15) | 0.270 |
| CXCL9 | 0.79 (0.86) | 0.69 (0.73) | 0.675 |
| SMPDL3A | -0.48 (0.77) | -0.68 (0.76) | 0.373 |
| EPHA1 | 0.16 (0.39) | 0.00 (0.48) | 0.200 |
| IFNGR1 | 0.35 (0.29) | 0.31 (0.44) | 0.639 |
| CTSC | 3.44 (0.71) | 3.65 (1.05) | 0.395 |
| CD276 | -0.11 (0.36) | -0.48 (0.68) | 0.015 |
| DBNL | 3.71 (2.91) | 1.94 (3.20) | 0.043 |
